# Developing a patient portal for haematology patients requires involvement of all stakeholders and a customised design, tailored to the individual needs

**DOI:** 10.1186/s12911-019-0868-y

**Published:** 2019-07-11

**Authors:** Paul A. F. Geerts, Trudy van der Weijden, Pien G. M. Loeffen, Lise E. F. Janssen, Celine Almekinders, Tobias A. Wienhold, Gerard M. J. Bos

**Affiliations:** 1 Department of Internal Medicine, Division of Haematology, School GROW, Maastricht University Medical Centre, P. Debyelaan 25, 6229 HX Maastricht, The Netherlands; 2 Department of Family Medicine, Maastricht University, School CAPHRI, Maastricht, The Netherlands; 3 Faculty of Health and Medicine, Maastricht University, Maastricht, The Netherlands

**Keywords:** Patient portals, Telemedicine, Haematologic neoplasms, Haematology, Neoplasms

## Abstract

**Background:**

Electronic patient portals are increasingly being implemented, also in (haemato) oncology. However, portal usage is low and depends on user and provider engagement. We explored wishes, expectations and thoughts of patients with a haematologic malignancy and their physicians with regard to the electronic patient portal.

**Methods:**

Based on insights from literature and a focus group discussion we built a 44-item questionnaire. This questionnaire was spread amongst patients with a haematologic malignancy at the outpatient clinic that was not yet exposed to patient portal facilities. Haematologists completed a questionnaire based on literature.

**Results:**

Patients were interested in many different types of access to information and portal functionalities. However, their opinions varied about the provision of access to the portal to other people, the role of the physician, possibilities for communication via the portal and timing of access. The physicians acknowledged the relevance of the electronic patient portal, but had some worries about the patients’ autonomous information handling, organizational and technical issues. Patients frequently expressed to be open about the potential of the patient portal to orchestrate their care. Nevertheless, most physicians appreciated their supporting role towards the patient.

**Conclusions:**

Patients and physicians appreciated the electronic patient portal. Both groups need to be involved in further portal development to improve engagement by meeting patients’ wishes, taking into account organizational and professional issues and managing expectations for both parties. To fit various patient profiles, portal design should be flexible and individualized. Further research should focus on the perceived added value and the impact on patient related outcome measures of portals.

**Electronic supplementary material:**

The online version of this article (10.1186/s12911-019-0868-y) contains supplementary material, which is available to authorized users.

## Background

The patients’ role in health care decisions is increasingly active and participatory, as illustrated by the implementation of shared decision making (SDM) [1]. By empowering patients they can make well-informed decisions. In the era of digitalization, an electronic patient portal can contribute to this empowerment. An electronic patient portal is “a website where patients can request their medical records, often supplemented with different options such as making an online appointment or getting a repeat recipe”. Patient portals are increasingly implemented; the Dutch minister of health even set a goal of 80% implementation in Dutch hospitals by 2019 [2]. Among selected groups of patients electronic patient portal implementation has already shown improvements in medication adherence, management of chronic disease, disease awareness, patient safety, improved patient experience, patient satisfaction and patient empowerment. An increase in preventative medicine and decrease in the number of office visits have also been reported [3, 4].

However, the use of patient portals is generally low [5]. Patients tend to use electronic Health (eHealth) services more when the development of the service has focused on the patients’ needs [5, 6]. Provider endorsement and engagement is also an important factor for patient engagement, as in most pre-portal studies physicians expressed various concerns about portal implementation [5, 6]. Therefore it is advised to involve both patients and health care providers in portal development.

Cancer patients, amongst whom patients with a haematologic malignancy, require frequent visits to their doctor, receive many lab results and are in need of various types of care. In cancer patients, the electronic patient portal adds to the autonomy, knowledge and psychosocial and behavioural skills of patients [7]. A recent example of patient involvement in portal development in cancer patients has been published in lung and breast cancer [8, 9] and publication of development methodology is awaited from a patient driven portal in chronic myeloid leukaemia [10]. A Canadian study showed that haemato-oncology patients were interested in using patient portals [11]. However, little is known regarding the preferences of haemato-oncology patients about the use and functions of the patient portal. Information about the health care providers’ view is limited in oncology [12–14] and absent in haemato-oncology.

The aim of this study is to explore the wishes, expectations and thoughts of patients with a haematologic malignancy and their physicians with regard to the electronic patient portal, in order to gain more support of patients and physicians for subsequent portal development. This may stimulate patient centred portal research and clinical development and, subsequently, empowerment in the haemato-oncology practice.

## Methods

### Study design

Exploratory sequential mixed methods design with two-step data acquisition [15]. First, by a focus group interview. Second, by a questionnaire survey. This method was chosen because the explorative character of the study required a qualitative aspect, while at the same time we aimed to explore a greater part of our patient population to increase the applicability of the results. This way the questionnaire results are better contextualised. Therefore, a patient questionnaire was based on literature research and a focus group discussion to explore relevant thoughts and opinions of the subject. A physician questionnaire was based on literature research.

### Participants

For the patient questionnaire survey, adult patients with a haematologic malignancy attending the haemato-oncology outpatient clinic at Maastricht University Medical Centre (MUMC+), an academic hospital in the Netherlands, were invited. The only exclusion criterion was not being able to read or write Dutch language. We aimed to collect data of 200 consecutive patients. Given the explorative character of the study and lack of comparable study results we were not able to calculate a sample size. We intended to include ample participants to analyse subgroups based on age (younger or older than 65) and gender.

For the physician questionnaire survey, all 14 haematologists and residents haematology of the MUMC+ were invited.

### The patient questionnaire

Literature research and a focus group discussion provided face validity of a questionnaire about patients’ preferences for electronic patient portal functionalities and considerations about using the electronic patient portal. The questionnaire consisted of two parts. First, demographic information was collected: age, gender, area of residence, education, access to Internet and if participants worked in health care. Also the control preference scale (CPS) single item was used [16, 17]. We hypothesized that these variables might impact the outcomes [11]. The second part consisted of 44 statements, divided in 9 themes. The statements were to be answered by a 5-point Likert scale ranging from no agreement to high agreement, and there was one open space for suggestions. The 9 themes were: access to different types of information, timing of access, availability of functionalities, communication, safety, providing (partial) access to other people, considerations about using the portal, worries about using the portal. See results table.

#### Literature research

We searched in PUBMED to explore existing electronic patient portals for haemato-oncology patients, the extent of patient involvement in developing these portals, what functionalities patients preferred for portals and barriers and facilitators for implementation of these portals. We searched by combining the following terms: “Hematologic Neoplasms”[Mesh], “Leukemia”[Mesh], “Lymphoma”[Mesh], “Multiple myeloma”[Mesh], electronic patient portal(s), patient portal(s), portal(s), and electronic health records. The search was restricted to articles starting from the year 1990, since Internet was not available for most people in the years before 1990 and electronic patient portals were not yet used. Also, the reference lists of these articles were scrolled and articles in the authors’ databases were used. Inclusion criteria were: (1) containing any information about electronic patient portal development, implementation or evaluation in any haemato-oncologic patient population; (2) English or Dutch language and (3) full text available. As this search yielded a limited amount of results, additionally, a comparable PUBMED search for existing electronic patient portals for oncology patients was performed to supplement the haemato-oncology literature.

#### Focus group

Seventy patients from the haemato-oncology outpatient clinic at the MUMC+ were asked by phone to volunteer in a focus group discussion, of which eight consented to participate and six eventually attended the focus group meeting. All volunteers had to be 18 years or older, suffered from a type of haematologic malignancy and were able to speak Dutch. Volunteers of different age and gender were recruited. After the volunteers had given verbal consent by phone, they received further information via an e-mail. The focus group took place in MUMC+ and was facilitated by two of the researchers (CA and LJ) as discussion leader and observer. After signed consent the discussion was started. The interview was structured by using an interview guide (Additional file 1), with discussion topics based on the literature research. The volunteers who attended the focus group received a small financial compensation including coverage for travel costs. The interview was audio recorded and transcribed verbatim by two researchers (TW and PL). Three researchers independently coded the transcript by thematic coding. Discrepancies were discussed between the three coding researchers and solved by consensus.

### The physician questionnaire

Literature research provided face validity of a questionnaire on physicians’ considerations about patients using the electronic patient portal. The questionnaire consisted of two parts. First, demographic information was collected: age, gender, staff function and experience working as haematologist. The second part consisted of 21 statements, to be answered by a 5-point Likert scale ranging from no agreement to high agreement, and one open space for suggestions. Also see the results table.

### Data collection and analysis

In October and November 2017 outpatient clinic employees approached approximately 250 consecutive patients to fill out the paper based questionnaire, directly before or after attending an outpatient clinic visit. In the same period, 320 patients, who were not approached for the paper based questionnaire, were sent an online version of the questionnaire by email. The age and gender of all patients attending the outpatient clinic in this period were registered. Also in the same period, the 14 physicians were invited by one of the researchers (PG) to fill out the physician questionnaire.

Outcomes of the demographic data were analysed descriptively by calculating means or frequencies. Age was also divided in groups (18–40, 41–50, 51–60, 61–70, 71–80 and above 80 years old). Gender and age groups of the participants were compared with the age of all patients by using the Chi Squared test. The Control Preference Scale was recalculated in three categories (autonomous, collaborative, passive) and analysed descriptively by calculating frequencies. Analysis with the Kolmogorov-Smirnov test determined that the questionnaire item responses were not normally distributed. The 5-point scale was recalculated in three categories (no agreement – neutral – agreement). The questionnaire items (5 and 3 category) were analysed descriptively by calculating frequencies. Subgroup differences between these frequencies were assessed by the Chi Squared test. For subgroups with more than two categories the Chi Squared test was performed, comparing each category with the others separately, to see which of the categories differed statistically significant. Consistency between items within each theme was tested with Cronbach’s alpha and factor analysis was performed, since after an interim analysis we hypothesized that a common construct might influence some of the answers. All calculations were performed with SPSS (SPSS statistics, version 23.0, IBM).

## Results

### Participants

Of the 570 patients that were invited, 204 (36%) agreed to participate. The quality of the response was satisfactory. There was a high response rate > 90% for all questionnaire items, but one item, that was completed by 87% of the responders. Of the 14 physicians that were approached, 13 responded and response to all questionnaire items was 100%.

The demographic and decisional role characteristics of the patients and physicians are shown in Tables 1 and 2.

**Table 1:** patient characteristics (*n* = 204)

Characteristic	Total
Median age (IQR) – yr.	63 (58–70)
Gender - %	
Male	64
Female	36
Residence - %	
Rural	45
City	55
Access to internet - %	
Yes	95
No	5
Employment in health care - %	
Yes	17
No	83
Type of questionnaire - %	
Paper	48
Digital	52
Control preferences scale, preferred role - %	
Autonomous	16
Collaborative	59
Passive	26
Education - %	
Low	36
Middle	28
High	37

**Table 2:** physician characteristics (*n* = 13)

Characteristic	Total
Median age (IQR) - yr.	35 (32–54)
Median work experience (IQR) - yr.	4 (2–16)
Gender - %	
Male	31
Female	69
Title - %	
Haematologist	62
Resident haematology	38

The study population age groups did not significantly differ from the total patient population, with exception of the 18–40 years old group that represented 16% of the total population and 5% of the study population (*P* < 0.001). Age was also not different (participants 64% and all patients 54% male, *P* = 0.2). Therefore we considered the study patients comparable for age and gender to the total patient population. Furthermore, the study population educational level was compared with the regional and national education level data as provided by the government. The study population well represented these characteristics. Additional patient characteristics can be found in Additional file 2.

### Patient questionnaire

The results for the 3-category items are shown in Table 3 and summarised below. The 5-category items did not add additional value to the results and are therefore not shown, they are available on request. Subgroup analyses revealed few relevant differences between groups, including the outcomes per type of questionnaire (paper or digital). We consider the implications of the differences in subgroups not relevant for the interpretation of our data because of the limited size of the differences, unless otherwise specified below. All statistically significant subgroup differences are shown in Additional file 3.

**Table 3:** patient questionnaire (responses in percentages)

Item	Disagree	Neutral	Agree
I would like to see the following about myself in the electronic patient portal			
1. Laboratory results	5	13	81
2. Imaging results	6	16	78
3. Medical patient file	4	10	86
4. Reporting to other physicians	5	13	82
5. Appointments in the hospital	3	5	91
6. Personal data	5	9	87
7. Current medication list	4	5	91
8. Medication history	5	14	81
I would like to have the following other functionalities in the patient portal			
9. Make appointments	4	19	77
10. Reminder for appointments	4	11	85
11. Request medication recipe	3	10	87
12. Change personal data	3	6	91
13. Make personal notes	15	32	53
14. Fill out questionnaires	6	25	69
15. Medication information	3	11	86
16. Disease information	4	16	80
17. Glossary of medical jargon	5	14	82
18. Patient organization information	6	28	66
I would like to see the information above (item 1–8) about myself in the portal at the following moment			
19. Directly when available	10	19	71
20. After my physician discussed them with me	7	12	82
I would like to have the following communication options			
21. With fellow patients	23	51	26
22. With allied health professionals	20	41	39
23. With my own physician	4	9	88
The following is important to me about patient portal safety			
24. Decide by myself about who gets access	7	8	85
25. Secure access	4	5	91
I would like to give the following persons full access to my patient portal, besides myself			
26. Partner	4	8	88
27. Volunteer caregiver	35	35	30
28. General practitioner	2	3	95
29. Other physicians in hospital	4	10	86
I would like to give the following persons partial access to my patient portal, besides myself			
30. Other physicians in hospital	3	8	89
31. Other health care professionals	19	26	56
32. Nurses	12	21	67
33. Apothecary	12	20	69
The following is important to me about the patient portal access			
34. Use it for clinic appointment preparation	4	16	81
35. See what my physician writes about me	4	13	83
36. Control my health care situation	3	14	83
37. Use it as a reference after a clinic appointment	4	9	88
38. I think it’s my right to see my results and file	3	14	84
39. Usage of plain language instead of physicians’ jargon	5	14	81
40. Information only opens after deliberately opening, not spontaneously	12	13	75
I have the following concerns about the patient portal			
41. Worry when I see results before the clinic appointment with my physician	33	16	51
42. Receive information I don’t understand without my physician’s help	25	15	60
43. The clinic appointment is focused more on portal details, instead of a personal conversation with my physician	26	25	50
44. My physician expects me to study my portal information before attending an appointment	26	18	56

Items 1 to 18: the large majority of patients (> 75%) would like to see or use 15 of the 18 proposed types of access to information and portal functionalities. A bit less robust, but still more than 50% was interested in the ability to take notes in the portal, to fill out questionnaires in the portal and to have access to patient organization information.

Items 19 and 20: more than two thirds of the patients would like to see the information as in items 1 to 8 both directly when it is available as well as after a physician consultation. We expected that patients would give a preference for either one of these two items, but more than half of the patients (55%) answered both items the same. Of the remaining 45%, the majority preferred immediate access to information to access after an appointment with their physician (*P* = 0.02).

Items 21 to 23: most patients were interested to contact their physician (88%) by the portal. Less than half of the patients would like to contact other patients or health care providers other than their own physician.

Items 24 to 33: almost all patients would like to decide by themselves who gets access to their portal. The patients varied in their preferences whom to provide access to their file. The great majority would like to give access to their partner, general practitioner and other physicians in the hospital. Patients were less interested to provide access to nurses, the pharmacist and allied health professionals and only 31% would like to give access to their volunteer caregiver.

Items 34 to 44: more than 75% of the patients agreed to the different motivations to use the patient portal. Also they would like physicians to use plain language instead of medical jargon. On the contrary, opinions were divided regarding possible concerns about the portal. Most noticeably, one third of the patients indicated that they would not be concerned about seeing their test results before the appointment with their physician, but more than half indicated that they would be concerned. Higher educated patients had less concerns than other patients, most pronounced in item 41 where almost half (48%) of the high educated patients disagreed as opposed to moderate (24%) and low (19%) educated patients.

### Physician questionnaire

The results of the 3-category items are shown in Table 4 and are described in summary below.

**Table 4:** physician questionnaire (response in percentages)

Item	Disagree	Neutral	Agree
The following is important to me about patients accessing the portal			
1. Patients study the portal information before attending an appointment	31	62	8
2. Patients can see what we write about them	15	54	31
3. Patients can have all results available to them	15	15	69
4. Patients can use the portal as reference after an appointment	15	0	85
5. It more actively involves patients with their treatment	0	23	77
6. I think patients have the right to see their data	0	15	85
7. Portal information is written in plain language without medical jargon	39	39	23
8. Patients can only open information deliberately	0	15	85
9. The information to patients only is available when results are definite	0	0	100
10. The patient can not see information before an appointment with a physician	0	0	100
11. The patient is able to contact the hospital more easy	15	31	54
12. The patient is able to contact other patients	31	39	31
I worry about the following, when patients will use the patient portal			
13. Patients get worried about accessing information before a physician appointment	0	8	92
14. Patients obtain information they don’t understand without the physicians’ support	0	0	100
15. The clinic appointment is focused more on portal details, instead of a personal conversation with the patient	8	15	77
16. The patient might feel obligated to study the patient portal before an appointment	46	39	15
17. The doctor-patient relationship might change	8	46	46
18. My workload might increase	15	31	54
19. I would get technical questions about the portal by patients	8	46	46
20. I might not respond soon enough to digital conversations and therefore the patient relationship changes	8	39	54
21. My schedule might have to change to account for patient portal activities	0	39	62

More than two thirds of the physicians acknowledged the importance of the ability for patients to access the portal to see test results, as a reference after an outpatient clinic appointment, to be more actively involved in their treatment and because they have the right to be able to access their patient file. They rarely agreed that patients would access the portal to prepare for an outpatient clinic appointment (8% agree) or to see what the physicians write about them in the patient file (31% agree).

Nearly all physicians felt that information should only be available after deliberately opening it, that only definite results should be available and that results should only be available to patients after a visit with the physician. Less than half of the physicians thought it is important for patients to contact other patients with the portal and about half to contact the hospital more easily. Only one physician agreed to report in the patient file in plain language, without jargon.

Physicians were concerned that patients might be anxious by seeing results before meeting the physician (92%) or without further explanation by the physician (100%). They we also concerned that by using the patient portal, outpatient clinic visits would be more focused on discussing details and questions regarding available biomedical results than on personal conversation on values and preferences. About half of the physicians were concerned that the portal could change the doctor-patient relationship. Few were concerned that a patient would feel obliged to prepare the outpatient clinic appointment by studying the portal information. Finally, physicians were moderately concerned about several logistic and technical issues.

## Discussion

### Overview

In this questionnaire study we assessed the wishes, expectations and thoughts of patients with a haematologic malignancy and of their haematologists regarding electronic patient portals. Due to the widespread rise of eHealth, many health care providers are under pressure to offer access to these portals. Unfortunately, portal utilization by patients has been generally low and one of the proposed solutions is to use participatory design approaches [5]. Participation starts with exploring preferences. Advances are being made in the field of (haemato) oncology and our questionnaire study complements previous work that has mainly been small-scale research. The questionnaire was applied before the electronic patient portal was available in the hospital under study, which provided us with an unbiased opinion of these patients.

### Main findings

Our study showed that more than 75% of patients were interested in being provided with various types of access and functionalities in an electronic patient portal. Nevertheless, at the same time patients’ opinions differed on various topics: the provision of access to the portal to other people, the role of the physician, possibilities for communication via the portal and timing of access. The physicians acknowledged a supporting role of the electronic patient portal, although they have some doubts and they still appreciate their own supporting role towards the patient.

### Study results in perspective

Previous studies in lung cancer, breast cancer, haematology and colorectal cancer showed similar interest of patients to portals as in our study [11, 13, 18, 19]. These studies were either small-scale or assessing only a limited amount of variables regarding electronic patient portals. Our study improves the scale and generalizability of these results.

Patients are clearly interested in using an electronic patient portal, but they vary in their individual preferences for practical use of the portal. Interestingly, these differences existed mostly throughout various subgroups of patients (age, gender, education), and less between these subgroups. A series of articles by Baudendistel and colleagues [12–14], where 14 patients with a colorectal malignancy were interviewed in 3 focus groups, confirms this variety of wishes, expectations and thoughts regarding electronic patient portal usage. This suggests there is not just one way of designing a portal for all patients and demands for customisation and flexibility of both developers and users.

Both physicians and patients think an electronic patient portal can empower patients in their health care situation, but from different viewpoints. The physicians in our study believe that patients need their help and guidance in understanding the information that is accessible in the portal, and worry about anxiousness when patients see this information without their help. In the study by Baudendistel, health care professionals were also concerned about patients autonomously handling information [14]. This is in line with findings in non-oncologic populations [5, 6, 20]. Interestingly, a quarter to a third of our patients does not expect to be anxious. A French study showed no mean difference in anxiety when patients gained access to a paper based medical file [21]. An evaluation of a patient portal implementation in a Dutch academic patient population showed that a minority of patients indicated that they did not like to see results on beforehand and would even be anxious about it [22]. The impact for these patients seeing their results could be large. This supports the need for customisation and flexibility of portals.

Furthermore, most patients indicated they preferred a glossary of medical jargon and the use of plain language instead of jargon by physicians. On the contrary, the physicians did not think information should be written in plain language. In another study by Baudendistel patients also expressed their wishes for a glossary [13]. Yet two other studies showed that such a reference library was only seldom used in practice [23, 24]. Having to write all medical notes in plain language would be a radical change of practice, moreover it would probably increase workload, which could be undesirable. Therefore, this topic requires further elaboration.

Indeed, patient portal implementation can also change the daily workflow, and possible workload, for health care employees. In our study, most physicians worried that their workload would increase. Baudendistel showed that health care professionals worry about receiving more messages by patients, when they get unrestricted information access [14]. Post-implementation, a MyChart study showed that only 5% of the activities originating from patient-to-healthcare messages were handled by physicians while the largest part was handled by nurses [25]. Thus, implementing a portal with communication functionalities will challenge not only physicians, but may even have more impact on the workload of other health care providers.

The mainly collaborative desired role in decision-making of our patients is noticeable. Previous studies in haematology populations in Germany and Australia showed that patients were more passive towards this role [26, 27]. Since shared decision-making has taken a rise in the past years, the results in our study might reflect a more active participation of patients in their healthcare management these days. Otherwise, the attitude of patients in different countries could differ. This might also influence eHealth preferences.

Finally, a small number of patients indicated that they did not have access to the Internet. So even in the current digital era, also these patients and their needs have to be taken into account.

Altogether, the above findings show that there is no ‘one-size-fits-all’ electronic patient portal. Since portal implementation is an intervention in daily clinical practice it requires an added value for patients and physicians to facilitate its actual adoption. A theoretical approach to further elaborate the added value is the capability approach, that states that people adopt an intervention when they perceive its empowerment [28, 29]. The variability of answers to the questions that assessed the motivations to use the portal suggest that some patients perceive a different added value of a portal than others. In order, this may require different portal functionalities for different patients. For example, a patient that values a thorough preparation of a clinic visit may need to see certain results timely and complete. While another patient who is anxious to see results in advance and who values a possibility to easily contact the clinic with questions afterwards, may need an easy method to communicate with the clinic. Therefore, developing a portal sets the developer and health care provider up for challenges: offering a broad range of possibilities, changing the current practice workflow and workload, and exploring where wishes and preferences meet the limit of current practice flexibility. This also means that expectations of users and professionals need to be managed, since not all expectations may be met.

### Strengths and limitations

The underpinning of the patient questionnaire items by literature and qualitative data emphasizes the robustness of our data. Our study was conducted in a haematology patient population in the Netherlands. Our results are complementary to earlier studies in the oncology field that have reported similar findings, and therefore we expect them to be applicable in a broader population than only haematology patients. Due to our fairly large sample size, we have been able to show that even within a relatively homogenous patient population there is variance of preferences.

Although the use of a 5-point Likert scale might reduce choice stress, it can also lead to a ceiling effect. We experienced a ceiling effect (many ‘complete agree’ responses) mainly on the variety of items assessing preferences for access and functionalities. This could imply that patients very clearly want to have all these different options. However, a large American study evaluating MyChart has shown that most patients only use a selection [24]. Factor analysis revealed that the responses in these categories were determined by a common construct. Therefore, our data cannot discriminate between these preferences and we prefer to conclude that patients are interested to use portal accessibilities and functionalities in general. Possibly, a discrete choice experiment could help to discriminate if this is desired.

The items assessing the timing of access to information in the portal also support that comparing items with each other is complicated. Although we expected patients to prefer one of both options, more than half of the patients answered these items the same. This might implicate that there is no fixed preference of access timing for the different types of information as in items 1 to 8. Another possibility is that patients did not relate the items to each other, which makes comparison less possible.

### Recommendations for practice

When health care providers are offering or planning to offer an electronic patient portal service, patients should be involved in the development, implementation and evaluation. Preferably different types of patients (acute and chronic care, cancer and non-cancer patients) should be involved.

Health care providers should also be involved in the development and implementation of the patient portal. Specifically, they can advice how the implementation of a patient portal could supplement the existing health care practice. This could ensure the continuing, though changing way of health care providers’ support to patients in the digital era.

The design of a portal should be customisable for each individual patient. Since patients will have different values and preferences, even within an apparently homogenous patient group as in our study, one should not limit the design of a patient portal to a single rigid format. This would make the portal more personal. For example, an introduction feature, exploring the individual patients’ preferences when first accessing the portal, and enabling tailoring to the individual patient’s needs, could be implemented. This would also require timely collaboration with information technology specialists to align the clinical wishes with technical availabilities.

### Recommendations for academia

Future research should confirm whether the above mentioned recommendations increase patient related outcome measures like patient satisfaction, quality of life, portal usage and empowerment, or health care associated outcomes like therapy adherence.

Furthermore, the perceived added value of patient portals to patients with a hematologic malignancy can be further explored, for example by using qualitative research methods. This may provide better understanding of the response variability to our questionnaire and subsequently improve further patient-centred portal development.

## Conclusions

Health care is continuously evolving and the digital revolution is an important development. Electronic patient portals are a major part of this. Our study shows that haematology patients are definitely open-minded to use an electronic patient portal. However, individual needs and preferences and the on-going involvement of health care providers urge portal developers to design these portals in a flexible, individualized way that fits various patient profiles. Our study results may help to develop more patient-centred portals with support from patients and physicians. Further research may focus on the perceived added value of patient portals and on the impact on patient related outcome measures of portal implementation.

## Additional files

Additional file 1: Interview guide. (DOCX 18 kb)

Additional file 2: Additional demographics. (DOCX 20 kb)

Additional file 3: Subgroups. (DOCX 20 kb)

## Data Availability

We supplemented the article with the most relevant data are within the additional files. All other data used and analysed during this study (e.g. SPSS files) are available from the corresponding author on reasonable request.

## Abbreviations

CPS: Control Preferences Scale
IQR: Interquartile range
MUMC+: Maastricht University Medical Centre +
SDM: Shared Decision Making
