# Supplementary material for: Developing a patient portal for haematology patients requires involvement of all stakeholders and a customised design, tailored to the individual needs
Source: BMC Med Inform Decis Mak. 2019 Jul 11;19:129. doi: 10.1186/s12911-019-0868-y (PMC6625061; doi:10.1186/s12911-019-0868-y)
Supplement: Supplementary file 1 — Interview guide. (DOCX 18 kb) [file 12911_2019_868_MOESM1_ESM.docx]

**Additional file 1: INTERVIEW GUIDE**

**Main questions** (with suggestion topics if no discussion rises):

Where do you think of, when you hear about a ‘patient portal’? (15 mins)

Do you have experience with using a patient portal in any other hospital? (5 mins)

What could a patient portal mean for you in practice? (20 mins)

Preparation for physician appointment

Better insight in health care situation

Practical issues

What information or functionality would you prefer in a portal? (20 mins)

Imaging

Lab results

Correspondence

Medical file

Medication

Questionnaires

Making appointments

Contact/communication

Email consults

**Backup questions in case of extra time**:

Do you have worries considering a portal

Anxiousness

Confrontation with disease

Privacy

Ambiguity when reading medical jargon

Who would you give access to your portal file?

Health care providers

Family

When would you like to see your results (e.g. lab)

Before or after clinic appointment

Influence on appointment preparation

What problems do you then expect?

Would you be disturbed by the use of medical jargon?
