# Supplementary material for: Developing a patient portal for haematology patients requires involvement of all stakeholders and a customised design, tailored to the individual needs
Source: BMC Med Inform Decis Mak. 2019 Jul 11;19:129. doi: 10.1186/s12911-019-0868-y (PMC6625061; doi:10.1186/s12911-019-0868-y)
Supplement: Supplementary file 2 — Additional demographics. (DOCX 20 kb) [file 12911_2019_868_MOESM2_ESM.docx]

**Additional file 2:ADDITIONAL DEMOGRAPHICS**

Table S1: Patient characteristics

|  | Characteristic | | Digital (%) | Paper (%) | All patients (%) |
| --- | --- | --- | --- | --- | --- |
|  | |  |  |  |  |
| **Gender** | |  |  |  |  |
|  | | Male | 70 | 58 | 64 |
|  | | Female | 30 | 42 | 36 |
| **Age** |  | |  |  |  |
|  | | <40 years | 6 | 4 | 5 |
|  | | 40-50 years | 7 | 4 | 5 |
|  | | 50-60 years | 29 | 18 | 24 |
|  | | 60-70 years | 36 | 44 | 40 |
|  | | 70-80 years | 21 | 22 | 21 |
|  | | >80 years | 2 | 7 | 5 |
| **Residence** |  | |  |  |  |
|  | | Rural | 43 | 46 | 45 |
|  | | City | 57 | 54 | 55 |
| **Highest education **** |  | |  |  |  |
|  | | None | 6 | 13 | 9 |
|  | | LBO, VBO, VBMO | 13 | 18 | 16 |
|  | | MAVO | 9 | 15 | 11 |
|  | | MBO | 24 | 21 | 22 |
|  | | HAVO, VWO, WO propedeuse | 8 | 3 | 6 |
|  | | HBO, WO bachelor | 26 | 26 | 26 |
|  | | WO doctoral, master | 16* | 5* | 11 |
| **Access to internet** |  | |  |  |  |
|  | | Yes | 95 | 91 | 93 |
|  | | Only through others | 3 | 4 | 3 |
|  | | No | 2 | 6 | 4 |
| **Working in healthcare** |  | |  |  |  |
|  | | Yes | 21 | 14 | 17 |
|  | | No | 79 | 86 | 83 |
| **Control**  **Preferences Scale** |  | |  |  |  |
|  | | Self | 5 | 0 | 2 |
|  | | Self, considering physician opinion | 12 | 15 | 13 |
|  | | Shared | 62 | 56 | 59 |
|  | | Physician, considering my opinion | 15 | 22 | 19 |
|  | | Physician | 7 | 7 | 7 |

* statistic significant difference between both types of questionnaire

** education levels are in the Dutch language and are displayed from the lowest (no education) to the highest level (doctoral or master degree) downwards.
