# Supplementary material for: Developing a patient portal for haematology patients requires involvement of all stakeholders and a customised design, tailored to the individual needs
Source: BMC Med Inform Decis Mak. 2019 Jul 11;19:129. doi: 10.1186/s12911-019-0868-y (PMC6625061; doi:10.1186/s12911-019-0868-y)
Supplement: Supplementary file 3 — Subgroups. (DOCX 20 kb) [file 12911_2019_868_MOESM3_ESM.docx]

**Additional file 3: SUBGROUPS**

For all items with statistical significance between the item responses between subgroups, frequencies are shown. All other items are not shown. Table S2 to S5 show these results for the categories with only 2 subgroup categories (binary subgroups). Table S6 and S7 show cross tables for the categories with 3 subgroup categories. For city/rural residence no significant differences were observed

Table S2: gender subgroups (response in percentages)

| Item | Male |  |  | Female |  |  |
| --- | --- | --- | --- | --- | --- | --- |
|  | Disagree | Neutral | Agree | Disagree | Neutral | Agree |
|  |  |  |  |  |  |  |
| 30 | 4 | 5 | 91 | 1 | 15 | 84 |
| 43 | 32 | 27 | 41 | 14 | 23 | 63 |

Table S3: health care employment subgroups (response in percentages)

| Item | Work(ed) in healthcare |  |  | Not work(ed) in healthcare |  |  |
| --- | --- | --- | --- | --- | --- | --- |
|  | Disagree | Neutral | Agree | Disagree | Neutral | Agree |
|  |  |  |  |  |  |  |
| 1 | 0 | 0 | 100 | 5 | 13 | 81 |
| 2 | 0 | 3 | 97 | 6 | 16 | 77 |
| 3 | 0 | 0 | 100 | 5 | 10 | 86 |
| 4 | 0 | 0 | 100 | 6 | 15 | 79 |
| 11 | 0 | 6 | 94 | 5 | 22 | 73 |
| 22 | 6 | 43 | 51 | 23 | 40 | 37 |
| 26 | 6 | 18 | 77 | 3 | 6 | 91 |
| 35 | 0 | 3 | 97 | 5 | 16 | 79 |
| 39 | 15 | 15 | 71 | 3 | 14 | 83 |

Table S4: questionnaire type subgroups (response in percentages)

| Item | Paper questionnaire |  |  | Digital questionnaire |  |  |
| --- | --- | --- | --- | --- | --- | --- |
|  | Disagree | Neutral | Agree | Disagree | Neutral | Agree |
|  |  |  |  |  |  |  |
| 3 | 8 | 7 | 85 | 1 | 12 | 87 |
| 4 | 9 | 14 | 77 | 2 | 12 | 86 |
| 6 | 8 | 8 | 84 | 1 | 10 | 90 |
| 8 | 8 | 10 | 81 | 2 | 18 | 80 |
| 11 | 9 | 17 | 75 | 0 | 20 | 80 |
| 13 | 7 | 9 | 85 | 0 | 11 | 89 |
| 14 | 6 | 6 | 89 | 0 | 7 | 93 |
| 17 | 7 | 9 | 85 | 0 | 13 | 87 |
| 20 | 10 | 21 | 70 | 3 | 35 | 62 |
| 21 | 32 | 48 | 20 | 15 | 54 | 31 |
| 22 | 30 | 37 | 33 | 11 | 44 | 45 |
| 23 | 7 | 11 | 82 | 1 | 7 | 92 |
| 25 | 8 | 4 | 88 | 1 | 5 | 94 |
| 26 | 7 | 12 | 82 | 2 | 5 | 93 |
| 35 | 9 | 13 | 78 | 0 | 14 | 87 |
| 36 | 7 | 18 | 76 | 0 | 12 | 89 |
| 38 | 4 | 7 | 89 | 1 | 20 | 79 |

Table S5: age subgroups (response in percentages)

| Item | <65 years old |  |  | 65 years and older |  |  |
| --- | --- | --- | --- | --- | --- | --- |
|  | Disagree | Neutral | Agree | Disagree | Neutral | Agree |
|  |  |  |  |  |  |  |
| 13 | 1 | 7 | 93 | 6 | 15 | 80 |
| 17 | 1 | 15 | 84 | 6 | 7 | 88 |
| 25 | 1 | 5 | 94 | 8 | 5 | 87 |
| 27 | 42 | 33 | 24 | 25 | 38 | 38 |
| 35 | 1 | 13 | 86 | 8 | 14 | 78 |
| 39 | 3 | 20 | 77 | 8 | 7 | 85 |

Table S6: control preferences scale subgroup differences (response in percentages)

|  | Characteristic | Disagree | Neutral | Agree |
| --- | --- | --- | --- | --- |
|  |  |  |  |  |
| **Item 9** |  |  |  |  |
|  | Autonomous* | 22 | 7 | 70 |
|  | Collaborative* | 5 | 17 | 78 |
|  | Passive | 11 | 27 | 62 |
| **Item 11** |  |  |  |  |
|  | Autonomous*^ | 7 | 0 | 93 |
|  | Collaborative*^#^ | 2 | 17 | 81 |
|  | Passive^^#^ | 6 | 31 | 63 |
| **Item 18** |  |  |  |  |
|  | Autonomous*^ | 7 | 0 | 93 |
|  | Collaborative*^#^ | 1 | 16 | 83 |
|  | Passive^^#^ | 6 | 25 | 69 |
| **Item 24** |  |  |  |  |
|  | Autonomous | 3 | 10 | 86 |
|  | Collaborative* | 6 | 3 | 92 |
|  | Passive* | 13 | 15 | 73 |
| **Item 26** |  |  |  |  |
|  | Autonomous*^ | 14 | 10 | 76 |
|  | Collaborative* | 3 | 6 | 92 |
|  | Passive^ | 0 | 13 | 87 |
| **Item 44** |  |  |  |  |
|  | Autonomous | 25 | 11 | 64 |
|  | Collaborative* | 25 | 26 | 50 |
|  | Passive* | 30 | 7 | 63 |

*^^#^ subgroups that statistically differ are marked in pairs.

Table S7: education subgroup differences (response in percentages)

|  | Characteristic | Disagree | Neutral | Agree |
| --- | --- | --- | --- | --- |
|  |  |  |  |  |
| **Item 1** |  |  |  |  |
|  | Low* | 7 | 20 | 73 |
|  | Moderate^ | 4 | 17 | 80 |
|  | High*^ | 3 | 3 | 94 |
| **Item 3** |  |  |  |  |
|  | Low* | 9 | 15 | 77 |
|  | Moderate^ | 2 | 13 | 85 |
|  | High*^ | 0 | 3 | 97 |
| **Item 4** |  |  |  |  |
|  | Low* | 6 | 18 | 77 |
|  | Moderate^ | 6 | 13 | 82 |
|  | High*^ | 0 | 6 | 94 |
| **Item 5** |  |  |  |  |
|  | Low* | 6 | 12 | 82 |
|  | Moderate | 2 | 6 | 93 |
|  | High* | 0 | 0 | 100 |
| **Item 6** |  |  |  |  |
|  | Low* | 6 | 18 | 77 |
|  | Moderate | 4 | 6 | 91 |
|  | High* | 1 | 4 | 94 |
| **Item 8** |  |  |  |  |
|  | Low* | 8 | 25 | 67 |
|  | Moderate | 4 | 13 | 83 |
|  | High* | 1 | 7 | 92 |
| **Item 14** |  |  |  |  |
|  | Low* | 6 | 9 | 84 |
|  | Moderate | 0 | 8 | 92 |
|  | High* | 0 | 3 | 97 |
| **Item 30** |  |  |  |  |
|  | Low* | 3 | 15 | 82 |
|  | Moderate | 4 | 6 | 90 |
|  | High* | 1 | 1 | 97 |
| **Item 35** |  |  |  |  |
|  | Low* | 11 | 15 | 74 |
|  | Moderate | 0 | 16 | 84 |
|  | High* | 0 | 10 | 90 |
| **Item 36** |  |  |  |  |
|  | Low* | 8 | 17 | 76 |
|  | Moderate | 0 | 16 | 84 |
|  | High* | 0 | 12 | 88 |
| **Item 37** |  |  |  |  |
|  | Low* | 8 | 15 | 77 |
|  | Moderate | 0 | 8 | 92 |
|  | High* | 1 | 3 | 96 |
| **Item 39** |  |  |  |  |
|  | Low | 6 | 9 | 85 |
|  | Moderate* | 0 | 8 | 92 |
|  | High* | 7 | 23 | 70 |
| **Item 41** |  |  |  |  |
|  | Low* | 19 | 24 | 57 |
|  | Moderate^ | 24 | 12 | 65 |
|  | High*^ | 48 | 12 | 41 |
| **Item 43** |  |  |  |  |
|  | Low* | 12 | 31 | 57 |
|  | Moderate | 25 | 23 | 52 |
|  | High* | 35 | 23 | 42 |

*^ subgroups that statistically differ are marked in pairs.
